# Application of Selenium Conjugated to Animal Protein in Laying Hens’ Diet for the Production of Selenium-Enriched Eggs

**DOI:** 10.3390/foods10061224

**Published:** 2021-05-28

**Authors:** Kai Qiu, Youbiao Ma, Uchechukwu Edna Obianwuna, Jing Wang, Haijun Zhang, Guanghai Qi, Shugeng Wu

**Affiliations:** Risk Assessment Laboratory of Feed Derived Factors to Animal Product Quality Safety of Ministry of Agriculture & Rural Affairs & National Engineering Research Center of Biological Feed, Institute of Feed Research, Chinese Academy of Agricultural Sciences, Beijing 100081, China; qiukai@caas.cn (K.Q.); mayoubiao@caas.cn (Y.M.); 2019y90100102@caas.cn (U.E.O.); wangjing@caas.cn (J.W.); zhanghaijun@caas.cn (H.Z.); qiguanghai@caas.cn (G.Q.)

**Keywords:** organic selenium, selenium-enriched egg, biosafety, antioxidative capacity, laying hen

## Abstract

The current experiment was conducted to investigate the application effects of selenium conjugated to insect protein (SCIP) in the production of selenium-enriched eggs. A total of 450 laying hens were randomly assigned to five dietary groups, each group consisting of six replicates. Hens in the control group received a diet without selenium supplementation, whereas hens in the other four groups received diets supplemented with either 1, 2, 5, or 10 mg/kg of selenium from SCIP. The productive performance, egg quality, antioxidant and immune capacity, biochemical indices, intestinal morphology, and oviduct health of laying hens were evaluated. The results showed that the supplementation of organic selenium provided by SCIP in the diets of laying hens enhanced performance and egg quality without any toxicity effect, even at the 10 mg/kg inclusion level. A level of 2 mg/kg of selenium provided by SCIP in diets tentatively improved the serum antioxidant and immune capacity, intestinal development, and oviduct health of laying hens in a conspicuous manner. Hence, the biosafety and positive effects of SCIP as a feed additive supplement in laying hens’ diet have been demonstrated with the enhanced production of safe and selenium-enriched eggs.

## 1. Introduction

Selenium is an essential nutritional trace element for many organisms, is involved in the formation of at least 25 selenoproteins, and enhances the antioxidant defense system of the body, thereby maintaining the normal physiology and optimal health of humans and animals [1].

Various studies have reported different health problems in animals due to selenium deficiency, including effects related to major metabolic functions, oxidant defense, the immune system, thyroid hormone metabolism, apoptosis, neurobiological functions, aging, and reproduction [2,3,4,5,6,7]. During commercial poultry production, birds are exposed to many stressors accruing from the environment, feeding and management, nutritional strategies, and physiological status [8], and in such stressful conditions, selenium requirement becomes substantially increased [1]. The potential of selenium in the nutritional modulation of the antioxidant capacity of birds, masking the effect of stressors in order to maintain poultry health and performance, has yet to be fully explored [9]. Nowadays, the dietary supplementation of selenium in various forms—sodium selenite, selenite, and nano-selenium, as well as organic forms of selenium, namely selenium yeast, selenium methionine (SeMet), OH-SeMet, and Zine-SeMet [10,11]—is common practice in the poultry industry in order to improve the immunity and overall health of the animals.

Though selenium at low dietary concentrations is essential for animal nutrition, inorganic selenium toxicosis appears when the concentrations are slightly above the normal range [12]. However, the expression of selenoprotein in the body, representing the bio-efficiency of selenium, is governed by two fundamental factors: selenium status and stress level [13]. Therefore, it becomes imperative to exploit non-toxic selenium sources as nutritional additives that can mitigate stressor effects on the birds during poultry production and overcome the problem of the optimal inclusion level. Optimal selenium supplementation is necessary not only for good poultry health but also to ensure the availability of quality selenium-enriched meat and eggs to consumers [14]. Previous studies have shown that clinical complications caused by prematurity in human infants could be minimized by selenium supplementation [15], and the intake of selenium was not enough to reach the reference values for patients with inflammation [16]. Long-term selenium supplementation offers a potential therapeutic effect on patients suffering from coronary artery disease [17]. Selenium also functions to prevent the expected metabolic alterations induced by physical inactivity and sedentary behaviors [18]. Selenium deficiency inhibits normal myocardial development and differentiation [5]. Therefore, it is necessary to increase selenium content in common human foods to improve overall health. Thus, selenium-enriched eggs have come into being.

A study by Marković in 2018 [19] revealed that increased intake of dietary selenium culminated in a corresponding increase of selenium content in eggs and meat. Organic selenium from selenium yeast or selenium-enriched kale sprout is more efficiently deposited into eggs than inorganic selenium from nano-selenium or sodium selenite in laying hens [20,21,22]. In order to increase the concentration of selenium and ensure safety without the toxic side effects of the diets, a new kind of organic selenium, selenium conjugated to insect protein (SCIP), was exploited for laying hens to produce safe selenium-enriched eggs. SCIP is obtained through two steps of biotransformation, microbial fermentation and insects synthesis, so it probably lends high bioavailability and biosafety and ensures a high biological value. In the current study, the effects of dietary SCIP on the productive performance, serum antioxidant and immune capacity, and intestinal health of laying hens were studied to investigate the application value and biosafety of SCIP on the production of selenium-enriched eggs.

## 2. Material and Methods

### 2.1. Ethics Statement

All experimental protocols were approved (AEC-CAAS-20191003) by the Animal Care and Use Committee of the Feed Research Institute of the Chinese Academy of Agricultural Sciences.

### 2.2. Test Material (Organic Selenium/Selenium Insect Powder) Production

To obtain reliable biosecurity, the organic selenium was produced through a series of procedures, as follows, including two steps of biotransformation. Firstly, using wheat bran and soybean meal as the raw materials supplemented with sodium selenite, selenium protein was synthesized by yeast fermentation as bacterial protein under optimal fermentation conditions. Secondly, the biosynthesized yeast selenium protein with a low selenium toxicity and a large dose tolerance range for insects, combined with vegetable and wheat bran, was fed to yellow mealworms to produce SCIP. Lastly, the SCIP was dried and ground into SCIP powder. The concentration of selenium in the SCIP powder used in the experiment was 4480 mg/kg. Meanwhile, a similar batch of insect protein powder without selenium was also prepared to balance the experimental diets.

### 2.3. Experimental Design and Bird Management

A total of 450 Hy-Line Brown laying hens (24-week-old, egg production rate = 94.0 ± 1.5%) were randomly allocated into 1 of 5 dietary treatments, with 6 replicates and 15 hens in each. The birds had free access to water and diets, and they were strictly managed according to the recommendations of Hy-Line International Online Management Guide (Hy-Line International, West Des Moines, IA, USA, 2011). Before the experiment, laying hens were fed a standard corn–soybean laying hen diet, which was adequate in all nutrients. The basal diet was formulated according to the nutrient requirements of the National Research Council (NRC, 1994) and Chinese Feeding Standard of Chicken (NY/T, 33-2004), and its ingredient composition and nutrient levels are shown in Table 1. The control group was fed basal diets without selenium supplementation. The treatment groups were fed basal diets supplemented with 1, 2, 5, or 10 mg/kg organic selenium provided by SCIP powder. Finally, the insect protein powder with no selenium was used to guarantee that all the experimental diets contained the same amount of insect protein powder. The calculated and analyzed selenium content in experimental diets are shown in Table 2. The pre-feeding trial lasted for 1 week to gradually acclimatize the birds to the change from the commercial laying hen diet to the experimental diets.

All birds were raised in three-tier battery cages with three birds in one cage (40 × 40 × 35 cm) in an environmentally controlled room with 16 h of light/day throughout the trial. The room temperature was maintained at 14–20 °C. During the 12-week trial, the egg number, egg weight, and mortality of each replicate were recorded on a daily basis. Feed consumption per replicate was recorded weekly. The birds were routinely vaccinated against bird flu and Newcastle disease at week 9. The average daily feed intake (ADFI), average egg production, average egg weight, and feed conversion ratio (FCR) during a given period were calculated based on the recorded data.

### 2.4. Sample Collection

At weeks 6 and 12 of the trial, ten eggs with weight closest to that of the replicate weight were selected. Five eggs were used for egg quality determination, while the other five were used for cholesterol (CHO) and selenium contents determination. One bird with the average BW of the replicate was selected per replicate for sample collection at weeks 6 and 12, and a total of 30 birds were selected each time. The selected birds were fasted for 12 h. Then, 5 mL of blood were collected from the wing vein and centrifuged at 3000 rpm/min for 15 min at 4 °C, and the obtained serum samples were stored at −20 °C until analysis.

At the end of week 12, the selected birds were euthanized with the intravenous use of pentobarbital sodium (100 mg/kg BW) and dissected under aseptic conditions. The liver, heart, and spleen of each bird were weighed, and their relative weight was calculated as the ratio of organ weight (g) to BW (g). The small intestine and oviduct were cut off and immediately placed on ice. The weight of the duodenum, jejunum, ileum, the whole small intestine, oviduct, and magnum was measured to calculate their relative weight index as tissue weight/BW × 1000‰, g/g. Additionally, after their mesentery was wiped off and they were straightened, their length was measured with a ruler. About 3 cm of the jejunum were gently cut off and fixed in 10% formalin for morphological analysis. One gram of oviduct tissue together with 2 mL of PBS were homogenized in an ice bath. The supernatant obtained through centrifugation at 4 °C and 12,000 rpm for 10 min was used for ELISA analysis.

### 2.5. Egg Quality Determination

Albumen and yolk were separated from the egg and weighed individually. After being dried naturally for three days and cleaned from albumen residuals, the eggshell was weighted. The ratio of albumen, yolk, and shell was calculated as their weight/egg weight ×100%. Eggshell thickness was measured using an Eggshell Thickness Gauge (ESTG1, Orka Technology Ltd., Ramat Hasharon, Israel) on three locations on the surface of eggs including the air cell, equator, and sharp end, the mean value of which was taken as the eggshell thickness. The eggshell-breaking strength measurement was conducted by an Egg Force Reader (Orka Technology Ltd., Ramat Hasharon, Israel). Albumen height, Haugh unit, and yolk color were detected using an Egg Analyzer (Orka Technology Ltd., Ramat Hasharon, Israel).

### 2.6. Histology and Morphometric Analysis of the Intestine

Intestinal villus height (VH) and crypt depth (CD) were measured in accordance with our previously reported method [23]. Fixed intestinal samples were dehydrated, embedded in paraffin wax, cut into serial 5 μm sections, and stained by hematoxylin and eosin. Histological sections were observed by a Nikon phase contrast microscope (Nikon Eclipse 80i, Nikon Co., Tokyo, Japan) at 40× magnification coupled with an integrated digital imaging analysis system. Five vertically crosscutting sections were selected for each sample. The vertical distances from the villous tip to villous–crypt junction level and from the villous–crypt junction to the lower limit of the crypt were taken as VH and CD, respectively. Ten well-orientated villi and their associated crypt per section were measured using an image analyzer (Lucia Software, Lucia, Za Drahou, Czechoslovakia). The mean value of 50 measurements per sample was submitted for statistical analysis.

### 2.7. Chemical Analysis

Serum biochemical parameters including glutamic amino transferase (ALT), aspartate amino transferase (AST), alkaline phosphatase (ALP), uric acid (UA), creatinine (CRE), and total bilirubin (TBIL) were detected with an automatic biochemical analyzer (Model 7020, Hitachi, Tokyo, Japan) using the corresponding kits with catalogue no. C009-2-1, C010-2-1, A059-2-1, C012-2-1, C011-2-1, and C019-1-1, respectively. The activities of glutathione peroxidase (GSH-px), superoxide dismutase (SOD), and total antioxidant capacity (T-AOC) in the serum were determined using the corresponding kits with catalogue no. A005-1-2, A001-3-2, and A015-1-2, respectively. The content of malondialdehyde (MDA) in the serum and yolk was measured using the kit with catalogue no A003-1-2. The contents of immunoglobulin A (IgA) and IgG in the serum and the contents of tumor necrosis factor α (TNF-α) and epidermal growth factor receptor (EGFR) in oviduct tissues were analyzed by the ELISA method using the corresponding kits (H108, H106, H052, and H032, respectively). All of the commercial kits were purchased from Nanjing Jiancheng Bioengineering Institute (Nanjing, China). Experimental procedures were strictly conducted according to the manufacturer’s instructions. The CHO content in yolk was detected by gas chromatography (TRACE 1300, Thermo Fisher Scientific, Rockford, IL, USA) with 5α-CHO (Sigma-Aldrich, Inc., Saint Louis, MO, USA) as an internal standard, based on the cholesterol determination method (GB/T9695.24-2008) published by the Standardization Administration of China. The content of selenium in experimental diets, whole egg, albumen, and yolk was determined by hydride-atomic fluorescence spectrometry (iCE 3300 AAS, Thermo Fisher Scientific, Rockford, IL, USA) coupled with a standard reference of selenium (GBW8551, National Sharing Platform for Reference Materials, China) according to the method (GB 5009.93-2017) published by the Standardization Administration of China.

### 2.8. Statistical Analysis

Experimental data were subjected to a one-way ANOVA or partitioned into linear and quadratic components using orthogonal polynomial contrasts and the GLM procedures of SAS 9.2 (SAS Inst. Inc., Cary, NC, USA) for a completely randomized design. *p* ≤ 0.05 was considered as statistical difference.

## 3. Results

### 3.1. Performance

The performance of the laying hens is shown in Table 3. The dietary supplementation of selenium had no significant effect on egg production and ADFI throughout the feeding trial compared to the control group. The FCR of laying hens during weeks 1–2 was significantly (*p* ≤ 0.05) decreased by dietary selenium supplementation, while no effect was observed in subsequent weeks to the end of trial. The FCR of laying hens in the whole trial period, weeks 1–12, was significantly different between dietary treatments (*p* = 0.05). Specifically, laying hens fed diets supplemented with 2 mg/kg of selenium showed a significantly (*p* ≤ 0.05) lower FCR compared to the control and 5 mg/kg selenium groups. Due to the routine vaccination at week 9, the laying rate and FCR of laying hens were negatively affected during weeks 9–10, but no differences emerged between groups. The average egg weight of the birds among the dietary treatments significantly differed during weeks 1–2, and that of those fed the diet supplemented 5 or 10 mg/kg of selenium was lower (*p* ≤ 0.05) than those fed 1 mg/kg of selenium. During weeks 5–6, the egg weight exhibited a quadratic change (*p* ≤ 0.05) as a decrease first and then an increase. The egg weight during weeks 3–4, 7–8, 9–10, 11–12, and 1–12 was not affected by dietary treatments. No more than two birds died in every group throughout the trial, and the data of mortality (not shown) did not show significant differences.

### 3.2. Egg Quality

As shown in Table 4, the albumen ratio of eggs at week 6 was significantly influenced (*p* ≤ 0.05) by dietary treatments and exhibited a quadratic change (*p* ≤ 0.05), first decreasing and then increasing along with the increasing of dietary selenium supplementation, but no differences were observed at week 12. The albumen height and Haugh units of eggs of laying hens fed 2 or 5 mg/kg of selenium were significantly (*p* ≤ 0.05) higher than those of the control group at week 12. The other indexes of egg quality including yolk ratio, shell ratio, egg shape, shell thickness, shell stiffness, shell strength, and yolk color at weeks 6 and 12 were not influenced by the dietary treatments. The contents of selenium, CHO, and MDA in eggs are listed in Table 5. At week 6, the selenium content in the whole egg showed linear and quadratic increases (*p* ≤ 0.05) along with the increase of dietary selenium supplementation, while the MDA content in yolk exhibited a quadratic decrease (*p* ≤ 0.05). The CHO content in the yolks of laying hens fed diets supplemented 1, 2, or 5 mg/kg of selenium was significantly (*p* ≤ 0.05) lower than the control group at week 6. The content of selenium in the whole egg, albumen, and yolk at week 12 showed linear and quadratic increases (*p* ≤ 0.05) as the dietary selenium supplementation increased. Dietary selenium had no effects on the contents of CHO and MDA in the yolk at week 12.

### 3.3. Biochemistry, Antioxidant Capacity, and Immune Indices of Serum

The effects of dietary selenium on the serum biochemical indices of laying hens are shown in Table 6. At week 6, the activity of AST in the serum of laying hens significantly differed among the dietary treatments, with that of the control group being higher (*p* ≤ 0.05) than the groups fed 1 or 2 mg/kg of selenium; at week 12, it exhibited a quadratic change (*p* ≤ 0.05) as an increase and then a decrease. The TBIL content in the serum was not different between treatments at week 6, but it significantly differed among treatments at week 12. Additionally, birds fed the 10 mg/kg selenium-supplemented diet had a higher (*p* ≤ 0.05) content of TBIL in the serum compared to those fed 2 or 5 mg/kg of selenium at week 12. The activities of ALT and ALP and the contents of UA and CRE were not influenced by dietary selenium supplementation at either week 6 or 12.

As shown in Table 7, at week 6, the activities of GSH-Px, SOD, T-AOC, and MDA content in the serum significantly differed *(p* ≤ 0.05) among the dietary treatments. In line with increased dietary selenium supplementation, the activities of GSH-Px, SOD, and T-AOC in the serum exhibited a quadratic change (*p* ≤ 0.05) as an increase followed by a decrease, while the content of MDA decreased first and then increased (*p* ≤ 0.05). The contents of IgG and IgA in the serum at week 6 were not affected by the dietary treatments. Following the trend of increased dietary selenium supplementation, at week 12, the activity of SOD in the serum showed a quadratic change (*p* ≤ 0.05), increasing and then decreasing, while the content of MDA exhibited the opposite quadratic change (*p* ≤ 0.05), decreasing and then increasing. The GSH-Px and T-AOC activities were not different among dietary treatments at week 12. The contents of IgG and IgA in the serum were significantly (*p* ≤ 0.05) influenced by dietary treatments at week 12. Specifically, the laying hens fed diets supplemented with 1 or 2 mg/kg of selenium had higher (*p* ≤ 0.05) contents of IgG and IgA than those fed the control diet.

### 3.4. Organ Indexes and Jejunum Morphology

**Figure 1:**
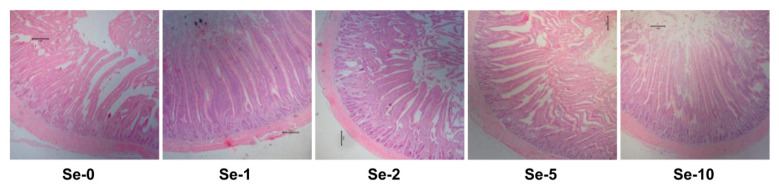
Effects of dietary selenium sources on the morphology of the jejunum in laying hens. The pictures (40×) are representative of the jejunum section with hematoxylin and erosion (HE) staining. Se-0: the birds fed the basal diet; Se-1, Se-2, Se-5, and Se-10: the birds fed 1, 2, 5, and 10 mg/kg of selenium provided by selenium-enriched insect protein powder, respectively.

The live body weight and the weight index of the liver, heart, and spleen were not affected by dietary treatments (Table 8). As shown in Table 9, the weight index of the intestine including the duodenum, jejunum, ileum, and the total small intestine was not influenced by dietary treatments. The length index of duodenum showed significant (*p* ≤ 0.05) quadratic changes, decreasing first and then increasing along with the increase of dietary selenium supplementation, while those of the jejunum, ileum, and the total small intestine were not affected. The representative sections stained by HE for jejunum morphology are shown in Figure 1, and the quantitative results are listed in Table 9. The VH of the jejunum increased first and then decreased, thus showing a significant quadratic change (*p* ≤ 0.05). The CD of the jejunum of laying hens fed 1, 2, and 10 mg/kg of selenium was significantly smaller (*p* ≤ 0.05) than that of the control group, and those fed 2 mg/kg of selenium had the smallest CD (*p* ≤ 0.05). The laying hens fed 2 mg/kg of selenium showed a higher V/C of the jejunum than the other groups (*p* ≤ 0.05).

### 3.5. Oviduct Development and Health

As shown in Table 10, the weight and length indexes of the oviduct and magnum were not influenced by dietary treatments. The oviduct tissues of laying hens fed 2 mg/kg of selenium showed a higher (*p* ≤ 0.05) protein expression of TNF-α than the others. The protein expression of EGFR exhibited linear and quadratic increases (*p* ≤ 0.05) with the dietary selenium supplementation increase.

## 4. Discussion

The supplementation of selenium in the diets of laying hens is critical not only to improve health status and performance but also to ensure the availability of high-quality products, such as selenium-enriched eggs, to consumers [14]. Positive effects of selenium on the performance of laying hens and the increased selenium contents in the eggs, along with increased selenium dietary supplementation within a certain range, has been demonstrated [13,19]. Due to the variation in tolerance threshold of laying hens to different forms of selenium [20,21,24] and the need to avoid the incidence of selenium toxicosis [12], it has become expedient to exploit non-toxic and high-bioavailability selenium sources as nutritional additives for laying hens.

Recently, various studies have investigated and confirmed the positive effects of different selenium sources including sodium selenite, nanoselenium, selenomethionine, and selenium yeast on the laying performance, egg selenium deposition, serum biochemical parameters, and antioxidant capacity of laying birds [20,21,22,25,26,27,28,29,30]. The effects of dietary source and concentration of selenium on broiler chickens have also been studied [31]. The productive process of the SCIP used in this study contains one more step of biological transformation than the usual organic selenium widely used in animal production at present, selenium yeast. The improvement of selenium on the performance of laying hens in the current study was verified again, in that adding 2 mg/kg of selenium provided by SCIP in the diet of laying hens significantly decreased the FCR. Though to 10 mg/kg of selenium was added into the laying hen diet as SCIP in this study, which was significantly greater than the levels of dietary selenium in previous reports but did not exceed the 0.5 mg/kg inclusion level [25,26,27,28], no treatment-related changes of toxicological significance were observed on performance and egg quality. Up to 3 mg/kg organic selenium from selenium-enriched yeast in diets was previously demonstrated to be without adverse effects for laying hens [30]. This was an indication that the organic selenium provided by SCIP has biosafety value, and, as such, selenium-enriched safe eggs became available to consumers. Under the condition that no selenium toxicosis occurs in laying birds, the addition of a high level of selenium in diets has led to the higher deposition of selenium in eggs [25,27,28]. In the present study, the selenium deposited into eggs including the yolk and albumen was significantly increased along with the increasing dietary SCIP supplementation, and the concentration of selenium in the yolk was more than 1 mg/kg after 12 weeks when dietary selenium was over 2 mg/kg. Therefore, it can be concluded that the biosafety of SCIP fully enabled the production of highly selenium-enriched eggs.

Due to health concerns concerning the relationship between dietary CHO and atherosclerotic cardiovascular risk, the consumption of eggs as a highly nutritious food was reduced because of the high CHO content in yolks [32]. MDA produced from polyunsaturated fatty acids by chemical reactions or enzyme-catalyzed reactions is the best investigated product of lipid peroxidation [33]. The oxidative stability of fresh eggs is reflected in the content of MDA in egg yolks [34]. In the current study, compared with the control group, laying hens fed the diet supplemented with 2 mg/kg of selenium showed significantly lower CHO and MDA contents in the egg yolk, and higher albumen height and Haugh units were observed in the eggs of diets with 1 or 2 mg/kg of selenium, which were consistent with the results of previous studies with selenium-enriched yeast [27,29]. Therefore, we deduced that the SCIP supplementation in the diet of laying hens could improve egg quality and probably extend shelf-life by increasing antioxidant capacity.

Various proteins or enzymes in the blood, such as AST, ALT, ALP, and TBILI, have been considered biochemical markers to assess hepatocyte inflammation and diagnose liver disease and liver injury [35,36,37,38]. In the present study, laying hens fed the diet supplemented with 1 or 2 mg/kg of selenium showed a lower content of AST in the serum, and those with 2 mg/kg of selenium also showed a relatively lower concentration of TBIL, which indicates that SCIP could improve liver function, probably because of the antioxidant property of selenium. This finding was not consistent with previous studies, which reported that dietary selenium showed no effects on the serum biochemical parameters of laying hens and rats [25,39] and the blood clinical parameters of laying hens [30]. This may have been due to the fact that the content of dietary selenium was higher than those in previous studies, which further highlights the biosafety of SCIP used in the current study.

GSH-Px, together with SOD and catalase, was considered to be the first line of antioxidant system of avian cells. Higher levels of selenium increase GSH-Px activity in the bodies of birds, since selenium is a key element in the structure of antioxidant enzymes [24,40]. Selenium supplementation in form of sodium selenite, nanoselenium, or selenium yeast in diets directly regulates GSH-Px activity by enhancing the GPx4 level, thus improving the antioxidant balance and development of laying birds [20,26,27]. In male albino mice exposed to fipronil, an insecticide, the reduced values of the antioxidant enzymes CAT and SOD were reinstated with selenium pretreatment, but such a counteracting effect was not observed in the control group [41]. The contents of T-AOC, T-SOD, and GSH-Px in the breast muscle of chicks were increased by selenium-enriched *Bacillus* sp. supplementation [42]. Selenium-enriched earthworm powder with 1 mg/kg of selenium improved antioxidative levels and immune function of laying hens by up-regulating the contents of GSH-Px, SOD, IgG, and IL-2 in the serum [43]. Dietary selenium increases the antioxidant levels and ATPase activity in the arteries and veins of poultry species [44]. In addition, the effects of sodium selenite and selenium yeast were found to be approximately equal in promoting antioxidant capacity of laying hens [45]. In the current study, laying hens supplemented with 1 or 2 mg/kg of selenium showed high activities of antioxidants enzymes (including GSH-Px, SOD, and T-AOC), a low content of MDA, and high contents of IgG and IgA in the serum, which was basically consistent with previous research and demonstrated that SCIP could improve the serum antioxidant and immune capacity of laying hens.

The dietary supplementation of selenium nanoparticles has been found to improve the gut function and development of broiler chickens [46,47]. In this study, laying hens fed the diet supplemented 2 mg/kg of selenium showed shallower CD and greater V/C values of the jejunum than those of the control group, which indicated that dietary SCIP improved the intestinal development of laying hens, probably because of the enhanced antioxidant effect of selenium on the intestine. TNF-α is an inflammatory cytokine produced by macrophages/monocytes during acute inflammation that is used by the TNF/TNFR cytokine superfamily to induce necrosis or apoptosis; is responsible for the maintenance and homeostasis of the immune system; influences inflammation and host defense; and is important for resistance to infection and cancers [48]. EGFR is a transmembrane glycoprotein, one of the four members of ErbB family of tyrosine kinase receptors, whose activation of EGFR is involved in regulating cellular proliferation, differentiation, and survival through a cascade of signaling pathways initiated by the autophosphorylation of receptor tyrosine kinase [49]. In the current study, the diet containing 2 mg/kg of selenium significantly increased the expression of TNF-α in the oviduct tissue of laying hens, and the protein expression of EGFR was also increased by diets with 2, 5, and 10 mg/kg of selenium. This was consistent with previous results that dietary selenium plays a protective role against aflatoxin B_1_- and ochratoxin A-induced inflammation and apoptosis in the liver and kidney [50,51], as well as selenium-deficiency resulted in the vein endothelial cell apoptosis of broiler chickens [7]. These results evidenced that a diet supplemented with 2 mg/kg of selenium from SCIP could improve the oviduct health of laying hens, which may be the biological pathway through which SCIP enhances performance and albumen quality.

## 5. Conclusions

Organic selenium provided by SCIP enhanced performance and egg quality while maintaining the optimal physiological status of birds, as indicated by normal gut morphology, magnum health, and enhanced biochemical, antioxidant, and immune indices. Safe and healthy eggs with low CHO, MDA, and selenium-enriched contents were guaranteed. In addition, an inclusion level of up to 10 mg/kg of selenium showed no toxic effects on the laying hens. This all highlights the biosafety of SCIP to be used as a feed additive in poultry diets, and an inclusion level of 2 mg/kg of selenium could be proposed as an optimum dose.

## Figures and Tables

**Table 1:** The composition and nutrient levels of the basal diet.

Ingredient, %	Contents	Nutrients ^1^	Contents
Corn	62.96	Crude protein, %	16.50
Soybean meal	26.41	Calcium, %	3.31
Limestone	8.80	Total phosphorus, %	0.54
Dicalcium phosphate	1.00	Available phosphorus, %	0.33
Salt	0.30	AME, MJ/kg	11.29
Insect protein powder	0.23	Lysine, %	0.86
Premix ^2^	0.30	Methionine, %	0.37
Total	100.00	Met + Cys, %	0.65

^1^ The values are calculated values. AME: apparent metabolizable energy. ^2^ Vitamin and mineral premix provided the following per kg of diets: VA: 12,500 IU; VD_3_: 4125 IU; VE: 15 IU; VK: 2 mg; VB_1_: 1 mg; VB_2_: 8.5 mg; VB_6_: 8 mg; VB_12_: 5 mg; calcium pantothenate: 50 mg; niacin: 32.5 mg; biotin: 2 mg; folic acid: 5 mg; choline: 500 mg; Mn: 65 mg; I: 1 mg; Fe: 60 mg; Cu: 8 mg; Zn: 66 mg; insect protein power: 250 mg.

**Table 2:** The selenium level of experimental diets.

Selenium, mg/kg	Se-0	Se-1	Se-2	Se-5	Se-10
Calculated value	0.08	1.08	2.08	5.08	10.08
Analyzed value	0.07	0.91	1.98	5.35	12.8

Se-0: the birds fed the basal diet; Se-1, Se-2, Se-5, and Se-10: the birds fed 1, 2, 5, and 10 mg/kg of selenium provided by selenium-enriched insect protein powder, respectively.

**Table 3:** Effects of dietary organic selenium on performance in laying hens.

Items ^1^	Se-0	Se-1	Se-2	Se-5	Se-10	SEM	*p*-Value ^2^		
							A	L	Q
Egg production, %									
Weeks 1–2	93.23	94.94	94.20	95.98	95.98	1.05	0.30	0.09	0.13
Weeks 3–4	92.78	95.00	94.29	92.30	92.62	1.43	0.62	0.39	0.67
Weeks 5–6	88.26	88.81	91.43	87.06	85.16	2.64	0.56	0.17	0.39
Weeks 7–8	91.90	91.48	94.37	90.32	89.44	1.60	0.27	0.13	0.32
Weeks 9–10	71.51	72.46	77.22	75.08	75.63	2.38	0.44	0.36	0.45
Weeks 11–12	89.37	90.79	92.22	91.51	89.52	1.73	0.73	0.67	0.51
Weeks 1–12	88.88	89.91	91.67	89.78	89.13	0.81	0.15	0.45	0.45
ADFI, g									
Weeks 1–2	117.81	117.78	114.92	116.90	116.16	1.04	0.28	0.47	0.67
Weeks 3–4	109.36	109.92	109.22	108.74	108.89	1.31	0.97	0.63	0.84
Weeks 5–6	113.40	115.24	114.06	114.71	112.99	0.91	0.41	0.32	0.33
Weeks 7–8	116.19	114.92	113.43	116.27	116.00	0.89	0.16	0.36	0.66
Weeks 9–10	116.47	117.47	118.17	117.68	117.53	0.89	0.75	0.73	0.69
Weeks 11–12	118.03	116.21	118.31	118.14	119.51	1.00	0.27	0.09	0.25
Weeks 1–12	116.52	116.06	115.96	116.71	116.21	0.61	0.90	0.95	0.92
FCR, g/g									
Weeks 1–2	2.19 ^a^	2.03 ^c^	2.05 ^c^	2.06 ^ab^	2.08 ^bc^	0.04	0.01	0.84	0.93
Weeks 3–4	2.01	1.96	1.96	2.02	1.99	0.05	0.84	0.78	0.90
Weeks 5–6	2.16	2.20	2.11	2.25	2.23	0.07	0.66	0.35	0.56
Weeks 7–8	2.09	2.11	2.01	2.16	2.14	0.05	0.25	0.24	0.46
Weeks 9–10	2.76	2.65	2.57	2.64	2.53	0.09	0.43	0.17	0.37
Weeks 11–12	2.18	2.08	2.11	2.14	2.18	0.04	0.39	0.38	0.54
Weeks 1–12	2.20 ^a^	2.15 ^ab^	2.12 ^b^	2.21 ^a^	2.18 ^ab^	0.02	0.05	0.62	0.81
Egg weight, g									
Weeks 1–2	58.24 ^abc^	59.44 ^a^	58.80 ^ab^	56.94 ^c^	57.90 ^bc^	0.55	0.04	0.13	0.08
Weeks 3–4	58.88	59.11	59.01	58.40	59.19	0.39	0.65	0.81	0.43
Weeks 5–6	59.90	59.04	59.12	58.77	59.85	0.35	0.11	0.46	0.04
Weeks 7–8	60.40	59.73	59.85	59.77	60.74	0.38	0.27	0.17	0.12
Weeks 9–10	59.73	60.62	59.74	59.77	60.73	0.34	0.09	0.14	0.88
Weeks 11–12	60.71	60.76	60.77	60.58	61.36	0.37	0.62	0.18	0.87
Weeks 1–12	59.61	59.75	59.53	58.99	59.89	0.26	0.18	0.62	0.06

^a,b,c^ Means within a row with no common superscripts significantly differ (*p* < 0.05). ^1^ Data are the mean of 8 replicates with 15 birds each; ADFI: average daily feed intake; FCR: feed conversion ratio (feed: egg, g:g); SEM: standard error of mean; Se-0: the birds fed the basal diet; Se-1, Se-2, Se-5, and Se-10: the birds fed 1, 2, 5, and 10 mg/kg of selenium provided by selenium-enriched insect protein powder, respectively. ^2^ A: ANOVA, the results of one-way ANOVA; L: linear, the results of linear regression analysis; Q: quadratic, the results of quadratic regression analysis.

**Table 4:** Effects of dietary organic selenium on the egg quality of laying hens.

Items ^1^	Se-0	Se-1	Se-2	Se-5	Se-10	SEM	*p*-Value ^2^		
							A	L	Q
Week 6									
Albumen ratio, %	64.70 ^ab^	65.22 ^b^	64.29 ^ac^	63.89 ^c^	65.30 ^ac^	0.28	0.03	0.12	0.03
Yolk ratio, %	24.48	23.97	24.92	25.06	24.86	0.30	0.11	0.18	0.14
Shell ratio, %	10.82	10.81	10.79	11.05	10.83	0.11	0.45	0.65	0.26
Egg shape index	1.33	1.32	1.32	1.32	1.33	0.01	0.90	0.71	0.84
Shell thickness, mm	0.46	0.46	0.45	0.46	0.46	0.01	0.91	0.87	0.70
Shell stiffness, N/mm	77.16	78.44	79.00	78.27	78.57	0.65	0.37	0.48	0.61
Shell strength, N	45.59	46.69	45.87	46.91	46.86	1.00	0.82	0.42	0.63
Albumen height, mm	7.46	7.54	7.50	7.54	7.41	0.20	0.99	0.72	0.87
Yolk color	5.58	5.73	5.43	5.43	5.63	0.17	0.66	0.94	0.56
Haugh units	86.11	86.62	86.80	86.62	85.68	1.25	0.97	0.61	0.78
Week 12									
Albumen ratio, %	64.39	65.26	64.43	64.32	65.12	0.40	0.23	0.27	0.28
Yolk ratio, %	25.33	24.83	25.36	25.46	24.70	0.39	0.55	0.37	0.40
Shell ratio, %	10.28	9.92	10.21	10.22	10.00	0.11	0.09	0.33	0.46
Egg shape index	1.32	1.33	1.32	1.32	1.33	0.01	0.90	0.66	0.79
Shell thickness, mm	0.46	0.46	0.46	0.47	0.45	0.01	0.26	0.72	0.13
Shell stiffness, N/mm	77.81	78.20	78.61	77.23	76.55	1.48	0.87	0.33	0.62
Shell strength, N	44.45	46.67	46.02	45.30	45.90	1.24	0.77	0.85	0.98
Albumen height, mm	7.39 ^b^	7.94 ^a^	7.91 ^a^	7.59 ^ab^	7.80 ^ab^	0.14	0.06	0.68	0.92
Yolk color	7.13	7.65	7.17	7.97	7.27	0.31	0.26	0.96	0.21
Haugh units	86.13 ^b^	89.15 ^a^	89.17 ^a^	87.18 ^ab^	88.10 ^ab^	0.83	0.07	0.91	0.98

^a,b,c^ Means within a row with no common superscripts significantly differ (*p* < 0.05). ^1^ Data are the mean of 8 replicates with 15 birds each; SEM: standard error of mean; Se-0: the birds fed the basal diet; Se-1, Se-2, Se-5, and Se-10: the birds fed 1, 2, 5, and 10 mg/kg of selenium provided by selenium-enriched insect protein powder, respectively. ^2^ A: ANOVA, the results of one-way ANOVA; L: linear, the results of linear regression analysis; Q: quadratic, the results of quadratic regression analysis.

**Table 5:** Effects of dietary organic selenium on the concentration of selenium, cholesterol, and malondialdehyde of eggs in laying hens.

Items ^1^	Se-0	Se-1	Se-2	Se-5	Se-10	SEM	*p*-Value ^2^		
							A	L	Q
Week 6									
Egg-selenium, µg/100 g	15.67 ^e^	29.67 ^d^	78.50 ^c^	80.67 ^b^	88.67 ^a^	0.52	<0.01	<0.01	<0.01
Yolk-CHO, mg/g	8.04 ^a^	6.32 ^c^	6.77 ^bc^	7.02 ^bc^	7.41 ^ab^	0.28	<0.01	0.59	0.29
Yolk-MDA, nmol/mL	88.70 ^a^	81.83 ^ab^	67.31 ^d^	70.38 ^cd^	77.83 ^bc^	2.72	<0.01	0.34	<0.01
Week 12									
Egg-selenium, µg/100 g	15.24 ^e^	31.94 ^d^	83.20 ^c^	85.13 ^b^	91.63 ^a^	0.55	<0.01	<0.01	<0.01
Albumen-selenium, µg/100 g	5.58 ^d^	11.00 ^c^	75.83 ^b^	76.83 ^b^	82.50 ^a^	0.44	<0.01	<0.01	<0.01
Yolk-selenium, µg/100 g	40.33 ^e^	86.33 ^d^	102.33 ^c^	106.67 ^b^	115.33 ^a^	1.39	<0.01	<0.01	<0.01
Yolk-CHO, mg/g	7.87	7.09	7.25	7.61	7.62	0.32	0.43	0.63	0.85
Yolk-MDA, nmol/mL	85.47	80.52	72.38	72.42	77.94	4.04	0.14	0.48	0.08

^a,b,c^^,d,e^ Means within a row with no common superscripts significantly differ (*p* < 0.05). ^1^ Data are the mean of 8 replicates with 15 birds each; CHO: cholesterol; MDA: malondialdehyde; SEM: standard error of mean; Se-0: the birds fed the basal diet; Se-1, Se-2, Se-5, and Se-10: the birds fed 1, 2, 5, and 10 mg/kg of selenium provided by selenium-enriched insect protein powder, respectively. ^2^ A: ANOVA, the results of one-way ANOVA; L: linear, the results of linear regression analysis; Q: quadratic, the results of quadratic regression analysis.

**Table 6:** Effects of dietary organic selenium on the blood biochemical indicators of laying hens.

Items ^1^	Se-0	Se-1	Se-2	Se-5	Se-10	SEM	*p*-Value ^2^		
							A	L	Q
Week 6									
ALT, U/L	6.81	7.01	6.93	7.08	6.69	0.14	0.34	0.29	0.13
AST, U/L	125.66 ^a^	119.34 ^b^	118.20 ^b^	120.90 ^ab^	121.26 ^ab^	1.69	0.05	0.81	0.43
ALP, U/L	343.68	325.89	339.08	320.41	338.40	8.55	0.29	0.97	0.24
UA, µmoL/L	161.44	157.19	154.07	157.32	159.16	2.89	0.49	0.82	0.59
CRE, µmoL/L	0.35	0.35	0.38	0.39	0.37	0.03	0.86	0.58	0.62
TBIL, µmoL/L	17.94	17.67	17.78	17.87	18.33	0.33	0.70	0.19	0.38
Week 12									
ALT, U/L	6.42	6.47	6.76	7.05	6.59	0.22	0.30	0.63	0.09
AST, U/L	118.35	117.37	118.69	120.58	118.99	0.75	0.07	0.22	0.04
ALP, U/L	355.52	356.18	346.37	344.13	365.82	8.46	0.40	0.29	0.15
UA, µmoL/L	160.46	158.58	154.49	156.32	159.12	2.74	0.57	0.89	0.46
CRE, µmoL/L	0.35	0.38	0.34	0.38	0.36	0.01	0.41	0.76	0.62
TBIL, µmoL/L	17.56 ^abc^	18.17 ^ab^	17.19 ^c^	17.46 ^bc^	18.24 ^a^	0.27	0.04	0.14	0.25

^a,b,c^ Means within a row with no common superscripts significantly differ (*p* < 0.05). ^1^ Data are the mean of 8 replicates with 15 birds each; ALT: glutamic amino transferase; AST: aspartate amino transferase; ALP: alkaline phosphatase; UA: uric acid; CRE: creatinine; TBIL: total bilirubin; SEM: standard error of mean; Se-0: the birds fed the basal diet; Se-1, Se-2, Se-5, and Se-10: the birds fed 1, 2, 5, and 10 mg/kg of selenium provided by selenium-enriched insect protein powder, respectively. ^2^ A: ANOVA, the results of one-way ANOVA; L: linear, the results of linear regression analysis; Q: quadratic, the results of quadratic regression analysis.

**Table 7:** Effects of dietary organic selenium on the serum antioxidant and immune capacity of laying hens.

Items ^1^	Se-0	Se-1	Se-2	Se-5	Se-10	SEM	*p*-Value ^2^		
							A	L	Q
Week 6									
GSH-Px, U/mL	744.75 ^b^	763.11 ^a^	766.34 ^a^	737.20 ^c^	735.87 ^c^	2.22	<0.01	<0.01	0.01
SOD, U/mL	177.18 ^c^	216.12 ^a^	217.42 ^a^	172.24 ^b^	164.18 ^c^	2.81	<0.01	<0.01	<0.01
T-AOC, U/mL	9.72 ^c^	10.55 ^a^	10.55 ^a^	9.57 ^b^	9.18 ^c^	0.13	<0.01	<0.01	0.02
MDA, nmol/mL	5.49 ^b^	4.52 ^c^	4.39 ^c^	5.55 ^a^	5.74 ^ab^	0.08	<0.01	<0.01	<0.01
IgG, g/L	4.40	4.35	4.31	4.28	4.41	0.05	0.35	0.59	0.13
IgA, g/L	2.20	2.18	2.16	2.11	2.21	0.04	0.46	0.76	0.16
Week 12									
GSH-Px, U/mL	723.75	756.91	751.55	726.60	729.75	21.28	0.72	0.59	0.86
SOD, U/mL	180.01 ^c^	205.34 ^a^	216.17 ^a^	203.45 ^bc^	187.11 ^ab^	6.24	<0.01	0.34	0.04
T-AOC, U/mL	9.04	10.58	10.07	10.67	9.38	0.50	0.11	0.63	0.10
MDA, nmol/mL	5.06 ^b^	4.82 ^b^	5.34 ^b^	6.08 ^a^	5.96 ^a^	0.19	<0.01	<0.01	<0.01
IgG, g/L	4.38 ^b^	5.12 ^a^	5.01 ^a^	4.50 ^b^	4.36 ^b^	0.13	<0.01	0.03	0.09
IgA, g/L	2.01 ^c^	2.19 ^ab^	2.24 ^a^	2.02 ^bc^	2.08 ^c^	0.05	0.01	0.45	0.74

^a,b,c^ Means within a row with no common superscripts significantly differ (*p* < 0.05). ^1^ Data are the mean of 8 replicates with 15 birds each; GSH-Px: glutathione peroxidase; SOD: superoxide dismutase; T-AOC: total antioxidant capacity; MDA: malondialdehyde; SEM: standard error of mean; Se-0: the birds fed the basal diet; Se-1, Se-2, Se-5, and Se-10: the birds fed 1, 2, 5, and 10 mg/kg of selenium provided by selenium-enriched insect protein powder, respectively. ^2^ A: ANOVA, the results of one-way ANOVA; L: linear, the results of linear regression analysis; Q: quadratic, the results of quadratic regression analysis.

**Table 8:** Effects of dietary organic selenium on the organ indexes of laying hens.

Items ^1^	Se-0	Se-1	Se-2	Se-5	Se-10	SEM	*p*-Value ^2^		
							A	L	Q
Liver, ‰	18.46	19.48	18.75	19.22	19.23	0.86	0.92	0.69	0.89
Heart, ‰	3.49	3.95	3.57	3.70	3.56	0.21	0.56	0.73	0.86
Spleen, ‰	1.47	1.54	1.30	1.33	1.30	0.12	0.54	0.26	0.42
Live body weight, kg	2.01	1.93	1.88	1.84	1.94	0.14	0.39	0.59	0.13

^1^ Data are the mean of 8 replicates with 15 birds each; the organ index was calculated as organ weight/live body weight. SEM: standard error of mean; Se-0: the birds fed the basal diet; Se-1, Se-2, Se-5, and Se-10: the birds fed 1, 2, 5, and 10 mg/kg of selenium provided by selenium-enriched insect protein powder, respectively. ^2^ A: ANOVA, the results of one-way ANOVA; L: linear, the results of linear regression analysis; Q: quadratic, the results of quadratic regression analysis.

**Table 9:** Effects of dietary organic selenium on intestinal development of laying hens.

Items ^1^	Se-0	Se-1	Se-2	Se-5	Se-10	SEM	*p*-Value ^2^		
							A	L	Q
Intestine index, ‰	33.15	34.14	31.51	31.81	34.66	2.68	0.90	0.65	0.68
Duodenum index, ‰	9.56	9.44	9.50	8.33	9.96	0.63	0.45	0.71	0.20
Jejunum index, ‰	13.15	14.57	11.37	12.92	13.45	1.37	0.59	0.90	0.86
Ileum index, ‰	9.04	10.12	9.67	10.56	11.25	1.01	0.95	0.45	0.75
Intestine length, mm	1391.67	1301.67	1380.33	1331.17	1385.00	44.46	0.54	0.68	0.68
Duodenum length, mm	120.67 ^ab^	109.33 ^b^	119.33 ^ab^	111.00 ^b^	127.00 ^a^	4.31	0.04	0.10	0.05
Jejunum length, mm	691.33	648.33	709.33	641.00	652.00	31.74	0.48	0.37	0.58
Ileum length, mm	579.67	544.00	551.67	579.17	606.00	22.26	0.32	0.08	0.21
VH, µm	1011.34	1055.95	1127.93	1174.06	1050.98	42.42	0.09	0.84	0.02
CD, µm	197.33 ^ab^	193.64 ^b^	165.53 ^c^	214.04 ^a^	185.76 ^b^	6.49	<0.01	0.94	0.38
V/C	5.15 ^b^	5.48 ^b^	6.84 ^a^	5.51 ^b^	5.66 ^b^	0.22	<0.01	0.93	0.68

^a,b,c^ Means within a row with no common superscripts significantly differ (*p* < 0.05). ^1^ Data are the mean of 8 replicates with 15 birds each; the organ index was calculated as organ weight/live body weight. VH: villus height of the jejunum; CD: crypt depth of the jejunum; V/C: the ratio of VH to CD of the jejunum; SEM: standard error of mean; Se-0: the birds fed the basal diet; Se-1, Se-2, Se-5, and Se-10: the birds fed 1, 2, 5, and 10 mg/kg of selenium provided by selenium-enriched insect protein powder, respectively. ^2^ A: ANOVA, the results of one-way ANOVA; L: linear, the results of linear regression analysis; Q: quadratic, the results of quadratic regression analysis.

**Table 10:** Effects of dietary organic selenium on oviduct health of laying hens.

Items ^1^	Se-0	Se-1	Se-2	Se-5	Se-10	SEM	*p*-Value ^2^		
							A	L	Q
Oviduct index, ‰	32.77	36.20	34.44	35.72	32.64	1.36	0.28	0.85	0.57
Oviduct length, mm	14.71	15.34	14.74	14.59	15.17	1.18	0.99	0.65	0.71
Magnum index, ‰	575	634	614	535	609	33.59	0.30	0.63	0.89
Magnum length, mm	318.33	346.40	335.67	302.33	322.00	17.44	0.50	0.49	0.53
TNF-α, pg/mg	3.04 ^b^	3.18 ^b^	3.86 ^a^	3.15 ^b^	3.23 ^b^	0.15	0.01	0.75	0.78
EGFR, ng/mg	0.69 ^b^	0.72 ^ab^	0.77 ^a^	0.82 ^a^	0.79 ^a^	0.03	0.02	0.04	<0.01

^a,b^ Means within a row with no common superscripts significantly differ (*p* < 0.05). ^1^ Data are the mean of 8 replicates with 15 birds each; the organ index was calculated as organ weight/live body weight. TNF-α: tumor necrosis factor α; EGFR: epidermal growth factor receptor; SEM: standard error of mean; Se-0: the birds fed the basal diet; Se-1, Se-2, Se-5, and Se-10: the birds fed 1, 2, 5, and 10 mg/kg of selenium provided by selenium-enriched insect protein powder, respectively. ^2^ A: ANOVA, the results of one-way ANOVA; L: linear, the results of linear regression analysis; Q: quadratic, the results of quadratic regression analysis.

## Data Availability

All data included in this study are available by contacting with the corresponding author.
